# The Impact of the COVID-19 Pandemic on Hepatobiliary and Pancreatic Surgical Services in Singapore: Retrospective Quantitative Study

**DOI:** 10.2196/29045

**Published:** 2022-05-23

**Authors:** Zhe Hao Timothy Teo, Cheong Wei Terence Huey, Jee Keem Low, Sameer Padmakumar Junnarkar, Vishalkumar G Shelat

**Affiliations:** 1 Department of General Surgery Tan Tock Seng Hospital Singapore Singapore; 2 Department of Hepatopancreatobiliary Surgery Tan Tock Seng Hospital Singapore Singapore

**Keywords:** audit, coronavirus, COVID-19, pandemic, surgery, impact, cancer, liver, pancreas, resource, elective surgery

## Abstract

**Background:**

At the height of the COVID-19 pandemic, the hepatopancreatobiliary (HPB) unit had to reorganize its surgical case volume due to the rationing of health care resources. We report on a local audit evaluating the impact of COVID-19 on the HPB unit and the HPB surgical oncology practice.

**Objective:**

The aim of this study was to review the impact of the COVID-19 pandemic on the HPB unit’s elective and emergency surgical cases. The secondary aims were to investigate the impact on the HPB surgical oncology operative case volume.

**Methods:**

We performed a comparative audit of the HPB unit surgical case volume for January-June 2019 (baseline) and 2020 (COVID-19). Elective and emergency cases performed under general anesthesia were audited. Elective cases included hernia and gallbladder operations and liver and pancreatic resections. Emergency cases included cholecystectomies and laparotomies performed for general surgical indications. We excluded endoscopies and procedures done under local anesthesia. The retrospective data collected during the 2 time periods were compared. This study was registered in the Chinese Clinical Trial Registry (ChiCTR2000040265).

**Results:**

The elective surgical case volume decreased by 41.8% (351 cases in 2019 compared to 204 cases in 2020) during the COVID-19 pandemic. The number of hernia operations decreased by 63.9% (155 in 2019 compared to 56 in 2020; *P*<.001) and cholecystectomies decreased by 40.1% (157 in 2019 compared to 94 in 2020; *P*=.83). The liver and pancreatic resection volume increased by 16.7% (30 cases in 2019 compared to 35 cases in 2020; *P*=.004) and 111.1% (9 cases in 2019 compared to 19 cases in 2020; *P*=.001), respectively. The emergency surgical workload decreased by 40.9% (193 cases in 2019 compared to 114 cases in 2020). The most significant reduction in the emergency workload was observed in March (41 to 23 cases, a 43.9% reduction; *P*=.94), April (35 to 8 cases, a 77.1% reduction; *P*=.01), and May (32 to 14 cases, a 56.3% reduction; *P*=.39); however, only April had a statistically significant reduction in workload (*P*=.01).

**Conclusions:**

The reallocation of resources due to the COVID-19 pandemic did not adversely impact elective HPB oncology work. With prudent measures in place, essential surgical services can be maintained during a pandemic.

**Trial Registration:**

Chinese Clinical Trial Registry (ChiCTR2000040265); https://tinyurl.com/ms9kpr6x

## Introduction

COVID-19 was first detected in Wuhan, China, in November 2019. Singapore registered its first COVID-19 case on January 23, 2020 [1]. Tan Tock Seng Hospital (TTSH) was designated as the main hospital to provide support to the National Centre for Infectious Diseases (NCID). TTSH is one of the largest tertiary hospitals in Singapore, with more than 1500 hospital beds. The surgical division of TTSH was tasked with augmenting the NCID workforce to handle the crisis.

The hepatopancreatobiliary (HPB) unit of TTSH started triaging and scaling down its operative and clinical workload as resources were reallocated preferentially for COVID-19–related care. International guidelines and opinion statements advocated for postponing non–oncology-related services, and thus, services for patients with benign gallbladder disorders and hernias were postponed for several months [2]. Despite the drop in overall case volume, it was possible to maintain essential surgical services in a pandemic situation through rapid situational audits to generate localized strategies [3]. Medication shortages, the unavailability of blood products and hospital beds, and delays for essential surgical procedures negatively impacted clinical care. Thus, many patients with non–COVID-19–related illnesses were not prioritized, and this could impact the quality and timeliness of care.

Rescheduling elective surgical operations became a norm to reduce the transmission of COVID-19 from hospital staff to the community and preserve hospital resources for patients with COVID-19 [4]. The general principle of triaging for elective surgical operations advocates a progressive reduction proportionate to pandemic escalation [5,6]. Triaging is critical to ensure fair, equitable, and just redistribution of public health resources. Professional societies and associations started providing guidance as evidence continued to emerge. The United Kingdom National Health Service published a guide listing surgical procedures and the suggested timeframe for each procedure [7]. Not all guidance will be generalizable across all health care systems, and hence, local policies remain integral.

Locally, it was agreed that the unit shall not compromise on the quality of care for patients without COVID-19, and thus, we established good practices like the index admission cholecystectomy policy, early operation for patients with pancreatic cancer and jaundice, and timely hepatic resections for resectable liver cancers continued to prevail. There are no data from HPB units about the impact of the COVID-19 pandemic on the provision of day-to-day services. We will be reporting our audit evaluating the impact of COVID-19 at its peak on our unit and its impact on HPB malignancy operations.

## Methods

### Ethical Considerations

This study was an audit of the effects of the COVID-19 outbreak on the HPB surgical unit and thus was exempt from ethics board review [8]. This study did not result in a departure from routine clinical care and no patient contact was made nor were any patient identifiers retrieved, stored, or disseminated.

### Background Information on the HPB Service

The HPB surgical service included 4 full-time faculty. Each faculty member was assigned to acute general surgery on-call duty 1 day per month and specialist HPB on-call duty 1 week per month. During pre–COVID-19 times, the service had 4.5 days allocated for procedures that required general anesthesia and shared a half-day list with other teams for local anesthesia procedures. According to the 2017-2018 audit, the unit had an annual case mix of 90 liver resections, 50 pancreatic resections, 350 biliary surgical procedures, 185 hernia procedures, and 90 unclassified major and minor procedures under general anesthesia. Biliary surgical procedures include elective and emergency cholecystectomy as well as complex biliary procedures. Hernia procedures include operations for inguinal, umbilical, and incisional hernias. Liver and pancreatic resections were routinely done as elective surgical operations. Cholecystectomy and hernia operations can be done either in an elective or emergency setting depending on the presenting symptoms of the patient. Elective surgical operations were defined as operations that can be scheduled in advance for medical conditions that were not immediately life-threatening. Emergency surgical operations were defined as operations that were done emergently, during the same admission, due to a potentially life-threatening medical condition.

We audited the surgical workload of the HPB team for the months of January-June and compared the workload in 2019 (baseline) with 2020 (COVID-19). The number of operations performed during these periods was collected from the surgeons' electronic case log. The different types of surgical procedures were sorted and classified based on the fixed charge code assigned to each operation. The retrospective data collected during the 2 time periods were compared. This study was registered in the Chinese Clinical Trial Registry (ChiCTR2000040265) and is reported in line with the Strengthening the Reporting of Cohort Studies in Surgery criteria [9]. We compared the workload between the years 2019 and 2020 using the chi-square test to examine differences, and *P* values <.05 were considered statistically significant. The primary aim of the study was to review the impact of the COVID-19 pandemic on the HPB unit’s elective and emergency surgical case volume. The secondary aims were to investigate the effects of the COVID-19 pandemic on the HPB surgical oncology services.

### General Precautions

The donning of surgical masks was mandatory within hospital premises. Twice daily temperature monitoring was compulsory and unwell staff were instructed to report to the occupational health clinic. All members of the HPB service were fitted for N95 masks and completed personal protective equipment (PPE) and powered air-purifying respirator training. To reduce the risk of COVID-19 transmission and minimize the impact on surgical services, the team was divided into 2 subteams to minimize interactions between members. Physical distancing among team members was reinforced continuously via audio broadcasts in the wards, email and WhatsApp chat group reminders, flyers in the elevator lobbies, and regular updates by senior hospital management. Nursing staff were appointed as safe distancing ambassadors and encouraged to flag any violations. Gatherings after ward rounds and during meals were stopped, and multidisciplinary meetings, journal clubs, and morbidity rounds were conducted remotely. The HPB service was segregated into 2 subteams with no cross-coverage at the registrar and consultant levels. Minimal cross-coverage among junior staff to sustain essential patient care activities was permitted to ensure compliance with junior physician duty hours. For example, if a junior physician covering team A is post call at noon, a junior physician from team B can provide basic coverage for patients under team A (eg, completing discharge documents and attending to nursing flags). Outpatient clinic lists were screened 2 weeks before the scheduled visit by the team consultants and registrars, and nonurgent cases were postponed. Cases were deemed nonurgent if patients were asymptomatic (eg, patients that were asymptomatic after biliary colic or hernia referrals). In addition, patients who were followed up after dyspepsia, surveillance endoscopies for intestinal metaplasia or colonic adenomatous polyps, liver cysts, cystic neoplasms of the pancreas, and so on, were seen via teleconsult and provided new appointments as per routine clinical care. Clinic patient lists were screened starting from April 7, 2020. The patient service associates followed up with new appointments and logistical inquiries. A script template was made available by senior management to ensure consistency in communication between health care workers and patients or next-of-kin.

### Precautions for Elective Surgical Operations

HPB multidisciplinary tumor board meetings remained operational, and these were conducted over a web-based platform with the radiologist, medical oncologist, and gastroenterologist. As staff were recruited to manage the NCID, a shortage of operation room nurses and anesthetists mandated a reduction in elective operation rooms. Thus, the operating lists were centralized and shared across the department. The committee consisted of the director of the operating rooms, the chief triage surgeon, and an anesthetist. Patients were categorized into groups A (procedures to be done in <2 weeks, eg, oncology), B (procedures to be done in <2 months, eg, symptomatic gallstones), and C (procedures that can be postponed by 3-6 months, eg, bariatric surgical operations). All listings were triaged weekly. Individual surgeons were permitted to approach the director of the operating rooms and the head of the department for expedited listing for justifiable reasons on a case-by-case basis. Chest x-rays were routinely done at the preoperative anesthetic clinic. Patients were informed that their elective operations could be cancelled and rescheduled if they tested positive for COVID-19. For suspected COVID-19 cases, the patients were isolated and the elective operation would only proceed after 2 negative nasopharyngeal swabs. An intubation-extubation protocol permits only the anesthetist and an assistant in full PPE (N95 mask, face shield, water-resistant gown, hairnet, and gloves) to be in the operating room during the intubation and extubation process and 5 minutes after. This protocol ensured that at least 2 cycles of gas exchange are completed and reduced COVID-19 transmission risk. Viral particles have been detected in the smoke generated by surgical energy devices used to cut and seal tissue during surgical procedures [10]. As COVID-19 transmission could occur due to the use of surgical energy devices, staff were made aware of this and educated via emails and circulars. The number of surgical team members was limited to 3 per case. The concentration of surgical smoke is higher in laparoscopic procedures than in open procedures. To mitigate this risk, we minimized the venting of smoke from trocars [11]. Further, a locally designed, 3D-printed, custom-made device was used to evacuate surgical smoke in a closed circuit.

### The Impact of the COVID-19 Pandemic

To free up critical care beds for patients with COVID-19, the division of surgery reduced the number of surgical intensive care unit (SICU) beds, high-dependency unit (HDU) beds, and general ward surgical beds. HDUs are intermediate care units between the SICU and general wards, where the ratio of nurses to patients (1:4) is higher than for general ward beds. HDU beds can support invasive monitoring (intraarterial catheters and central venous pressure monitoring) and noninvasive ventilatory support. SICU beds offer a 1:2 ratio of nurses to patients and invasive support, such as endotracheal intubation and continuous renal replacement therapy. HDU beds were reduced from 28 to 10 and SICU beds were reduced from 10 to 8. Thus, the number of major elective general surgery procedures had to be reduced. As our hospital hosted the NCID, staff and resources were diverted to upscale the clinical needs of the NCID to combat COVID-19. As a result, polyclinic and general practice referrals were diverted to other hospitals, selected ambulance cases were diverted to nearby hospitals, and all listed surgical patients were triaged by the central committee. Further, the segregation of designated “clean” and “dirty” surgical teams was done to reduce the risk of health care transmission and ensured a critical specialist pool was available all day and night to sustain essential services, including HPB oncology.

## Results

Our unit performed 351 elective surgical procedures from January-June 2019. The procedures included 155 hernia repairs, 157 gallbladder operations, 30 liver resections, and 9 pancreatic resections. A total of 204 elective surgical procedures were performed from January-June 2020. This included 56 hernia repairs, 94 gallbladder operations, 35 liver operations, and 19 pancreatic resections (Table 1). In 2020, all patients with pancreatic resection had malignancies, and the majority of patients who underwent liver operations had malignancies (30/35, 86%). Of the 5 patients with benign liver disease, 3 had symptomatic giant liver cysts and underwent cyst deroofing operations, 1 patient was offered an operation for recurrent pyogenic cholangitis due to multiple episodes of sepsis, and the last patient had a pyogenic liver abscess masquerading as hepatocellular carcinoma (HCC). The gallbladder operations were mainly done laparoscopically. The pancreatic resections were done using an open approach, except for 2 laparoscopic distal pancreatectomies. Of the 35 liver resections, 17 (49%) were done laparoscopically. The number of hernia operations decreased by 63.9% (155 in 2019 compared to 56 in 2020; *P*<.001) and cholecystectomies decreased by 40.1% (157 in 2019 compared to 94 in 2020; *P*=.83). The liver and pancreatic resection volume increased by 16.7% (30 cases in 2019 compared to 35 cases in 2020; *P*=.004) and 111.1% (9 cases in 2019 compared to 19 cases in 2020; *P*=.001), respectively.

**Figure 1:**
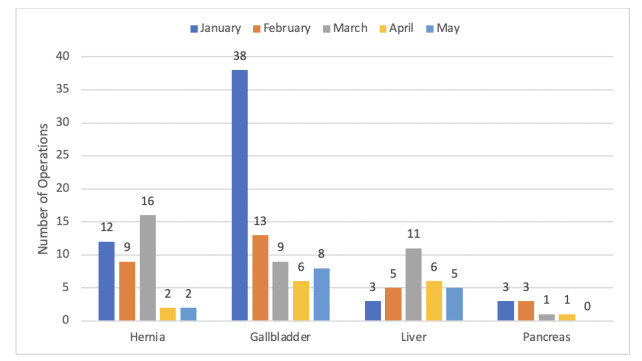
Elective procedures performed by the hepatopancreatobiliary unit in January-June 2020.

Figure 1 shows the elective surgical workload from January-June 2020. We did not operate on any patients with an active or past diagnosis of COVID-19. The reduction in case volume for gallbladder operations started in February 2020 and continued until April 2020, after which it returned to a “new normal,” and it is still well below the expected baseline state. Similarly, the reduction in caseload for hernia operations started in April 2020 and has reached the baseline rates from June 2020. The liver and pancreatic procedure volume did not decrease.

**Figure 2:**
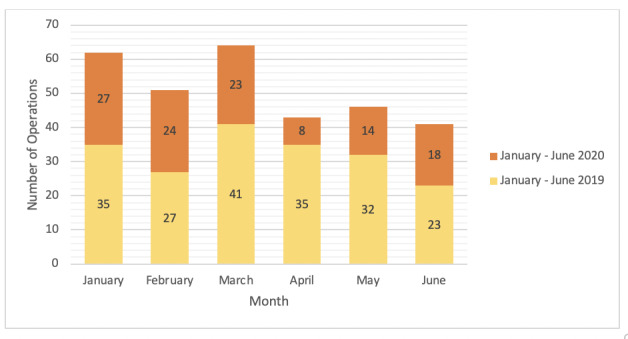
Emergency procedures performed by the hepatopancreatobiliary unit in January-June 2019 and 2020.

Figure 2 shows the emergency surgical procedures from January-June in 2019 (n=193) and 2020 (n=114). Emergency cases included superficial abscesses, acute appendicitis, acute cholecystitis, obstructed hernias, obstructed small or large bowel tumors, and perforated intraabdominal viscera. The emergency surgical workload reduced from 193 to 114 cases (40.9% reduction). The most significant reduction in the emergency surgical workload was observed in March (41 to 23 cases, 43.9% reduction; *P*=.94), April (35 to 8 cases, 77.1% reduction; *P*=.01), and May (32 to 14 cases, 56.3% reduction; *P*=.39); however, only April had a statistically significant reduction in workload (*P*=.01).

**Table 1:** A comparison of the characteristics of elective surgical operations done in 2019 (n=351) and 2020 (n=204).

Type of elective operation		January-June 2019, n (%)^a^	January-June 2020, n (%)^a^
**Liver operation^b^**			
	Benign liver pathology	1 (3.3)	5 (14.3)
	Minor resection (<2 segments)	21 (70)	17 (48.6)
	Major resection (>3 segments)	8 (26.7)	13 (37.1)
**Pancreatic resection^c^**			
	Distal pancreatectomy	1 (11.1)	2 (10.5)
	Whipple or total pancreatectomy	8 (88.9)	17 (89.5)
**Gallbladder operation^d^**			
	Laparoscopic cholecystectomy	137 (87.2)	80 (85.1)
	Open cholecystectomy	7 (4.5)	6 (6.4)
	Cholecystectomy with common bile duct exploration	13 (8.3)	8 (8.5)
**Hernia repair^e^**			
	Laparoscopic hernia repair	82 (52.9)	32 (57.1)
	Open hernia repair	73 (47.1)	24 (42.9)

^a^Percentages are calculated based on the category totals for each year.

^b^This category comprised 8.5% (30/351) of elective operations in 2019 and 17.2% (35/204) of elective operations in 2020; *P*=.004.

^c^This category comprised 2.6% (9/351) of elective operations in 2019 and 9.3% (19/204) of elective operations in 2020; *P*=.001.

^d^This category comprised 44.7% (157/351) of elective operations in 2019 and 46.1% (94/204) of elective operations in 2020; *P*=.83.

^e^This category comprised 44.2% (155/351) of elective operations in 2019 and 27.5% (56/204) of elective operations in 2020; *P*<.001.

## Discussion

### Principal Findings

The reallocation of resources during the COVID-19 pandemic impacted our elective benign surgical work and emergency surgical services but did not adversely impact elective HPB oncology work. We observed an increase in the HPB oncology volume during the COVID-19 pandemic. These findings emphasize that measures taken for resource reallocation were appropriate and fulfill the principles of distributive justice in public health ethics.

Locally, many measures were implemented to ensure that patients who needed urgent, prompt, or early elective surgical procedures were not deprived of this opportunity by having their procedures canceled or postponed due to the COVID-19 pandemic. As the pandemic escalated, designated individual operating lists were reduced, and lists were made communal for the whole surgical division. Our results showed that with the previously mentioned framework in place, essential surgical operations can still be prioritized in a pandemic.

We witnessed a substantial drop in our elective cholecystectomy and hernia workload, and this was attributed to the success of our triaging system. There was a nonstatistically significant reduction in the cholecystectomy workload, partly as symptomatic gallstone disease was the primary contributor to our unit’s workload. The most significant impact of triaging was observed in the inguinal hernia procedure workload (*P*<.001). By postponing nonurgent elective cases, the HPB unit continued to provide timely and adequate services for patients with HPB malignancies. We witnessed a paradoxical increase in the volume of both liver (*P*=.004) and pancreatic (*P*=.001) resections. This phenomenon has not been reported before and merits discussion.

### Elective HPB Malignancy Cases

The increase in HPB oncology volume was an interesting result from this study. The underlying reasons for this were multifactorial. In pre–COVID-19 times, patients with symptomatic gallstones and hernias competed for slots on the operating room lists. During the COVID-19 pandemic, due to effective triaging mechanisms, even though overall resources were reduced, the relative number of resources increased for patients with HPB cancers. Another contributing factor could be the placement of patients with HPB cancers on the waiting list as priority was given to this group of patients. Potentially, this phenomenon may not be observed in low-volume HPB units with shorter waiting times or geographies with low disease prevalence for HCC and pancreatic cancer. Locally, the disease burden of HCC is substantial due to the high seroprevalence of the hepatitis B virus and the emergence of nonalcoholic fatty liver disease [12]. Unlike breast cancers, there are no effective neoadjuvant chemotherapy or hormone therapy treatments, and hence delaying operations can impact survivorship [13-15]. In patients with pancreatic cancer or cholangiocarcinoma presenting with obstructive jaundice, emergency operation is crucial in obtaining curative treatment and preventing the development of cholangitis. Sud et al [16] investigated the impact of surgical delays on survival for different cancers and estimated that a delay in operation for pancreatic cancers could lead to >30% reduction in survival at 6 months and >17% reduction in survival at 3 months. The American College of Surgeons, Society of Surgical Oncology, and European Society of Medical Oncology have provided inpatient management guidelines for patients with HPB cancers that are in line with our stance to offer surgical operations in oncologically resectable cases [6,13,17].

### Elective Nonmalignancy Cases

We continued to prioritize patients with symptoms of groin pain or recurrent attacks of biliary colic to reduce the impact on quality of life and maintain good patient-reported outcomes [18,19]. The number of first outpatient visits to surgical clinics for new patients decreased by about one-half. This ensured that only symptomatic patients were referred to the clinics. As the local cases of COVID-19 came under control, restrictions were eased, and the number of cases of cholecystectomies and hernias increased in June 2020. The cholecystectomy workload has not recovered to that of pre–COVID-19 times. This could be due to a fear of visiting public hospitals and seeking treatment at private health care facilities among patients. As the outpatient referral pattern resumes slowly, we expect the workload for hernia and gallbladder surgical operations to be restored gradually.

### Emergency Cases

In our experience, the emergency workload reduced gradually. From March 2020, the effect was significant due to hospital guidance for complete ambulance diversion for non–COVID-19–related illnesses. This trend continued during the ensuing months. Further, the “circuit breaker” period of April-May 2020 also resulted in a reduction in the number of walk-in patients in the emergency department. Surgical patients suspected to have COVID-19 were isolated and required to have nasopharyngeal COVID-19 swabs done. Patients with pending swabs that required lifesaving surgical procedures were operated on with full PPE in a specialized operating room designed for COVID-19 operations. Patients with superficial abscesses and patients who were hemodynamically stable were managed with antibiotics first until their swabs were negative. Patient investigations and management were not delayed under the pretext of COVID-19. We did not change the local policy of managing acute appendicitis, acute cholecystitis, hollow viscus perforation, or obstructed hernia due to the COVID-19 pandemic.

### Strengths and Limitations

Strict adherence to triaging guidelines was essential to ensure the appropriate allocation of limited resources. Due to the nature of the triaging system, we had to be flexible with our schedule to perform the essential surgical operations. We had a team coordinator who was responsible for informing patients of the timing of their operations and ensured that the required preoperative workups were arranged. We created a separate list of patients who were willing to come in for their operations on short notice and thus managed to fill in the empty slots if there were cancellations. Memorial Sloan Kettering Cancer Center in the United States had a similar framework to ours for triaging patients for “essential” cancer operations, and they had managed to continue operating on patients with cancer even as COVID-19 cases escalated in the United States [20].

There were a few limitations to our audit. Firstly, we did not collect hospital-wide admissions data and reported only the HPB unit data as a department-wide audit would introduce heterogeneity. For example, the colorectal surgical service had minimal elective hernia and gallbladder volume, and vascular surgical services do not participate in emergency or elective general surgical pathologies. Secondly, we did not report on the clinical outcomes of patients as the primary intent was to audit the impact of COVID-19 on the service and not to report clinical outcomes This is also one reason why we did not submit this study for ethical approval. Lastly, we did not conduct audits on the availability of blood products, medications, and intensive care beds. In a multicenter study including 34 pediatric oncology centers from 19 countries, Saab et al [21] reported that essential treatments were delayed, and certain centers had shortages of blood products, beds, and medications. However, we did not observe any cancellations due to logistics or resource issues.

### Conclusions

**Figure 3:**
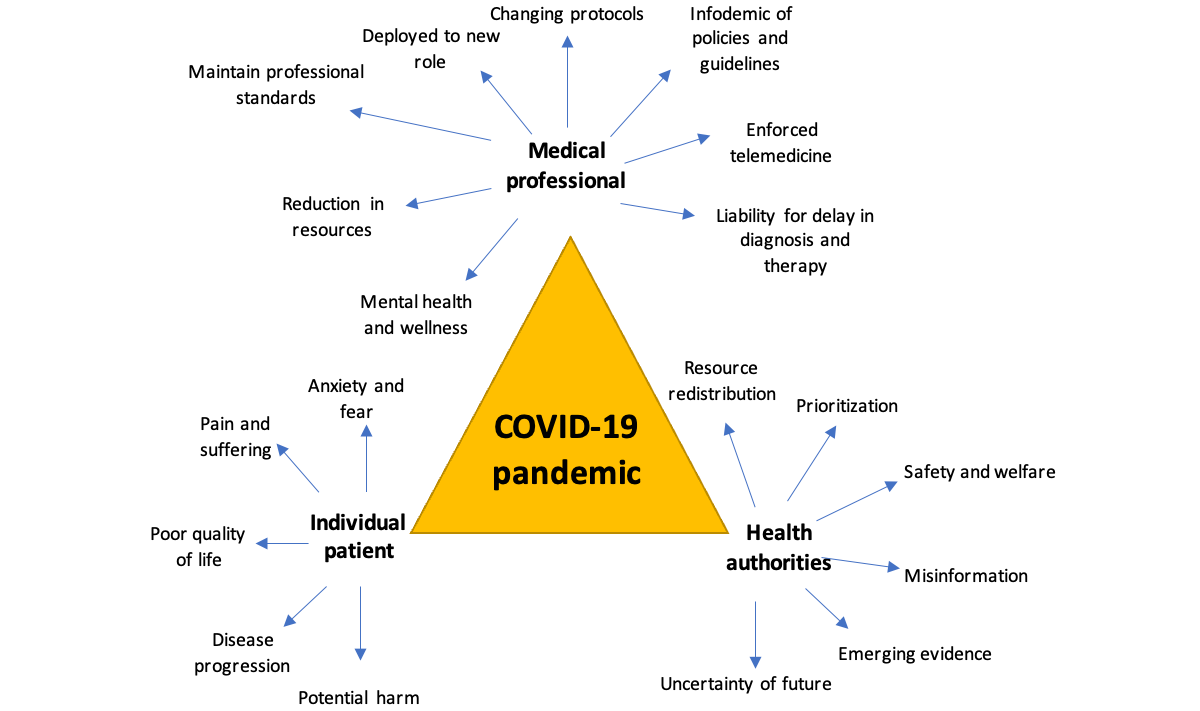
Implications for stakeholders during the COVID-19 pandemic.

The COVID-19 pandemic will continue for the months ahead [22], and our audit reassures various stakeholders (Figure 3) that the measures implemented locally remain valid and proportionally appropriate to maintain the functionality of the HPB surgical oncology service. Our results have shown that with a well-organized protocol in place, essential surgical procedures can still proceed in a pandemic despite resource reallocation and rationing.

## Abbreviations

HCC: hepatocellular carcinoma
HDU: high-dependency unit
HPB: hepatopancreatobiliary
NCID: National Centre for Infectious Diseases
PPE: personal protective equipment
SICU: surgical intensive care unit
TTSH: Tan Tock Seng Hospital

## References

[1] Past updates on COVID-19 local situation. Ministry of Health Singapore.

[2] Jie TTZ, Junnarkar SP, Shelat VG (2021). Pluralibacter georgoviae Causing Acute Cholecystitis with Perforation. Surg Infect (Larchmt).

[3] Omar UF, Pei Yein T, Rajaratnam V (2020). Managing hand and reconstructive microsurgery service during COVID-19 pandemic: Singapore experience. Postgrad Med J.

[4] Moletta L, Pierobon ES, Capovilla G, Costantini M, Salvador R, Merigliano S, Valmasoni M (2020). International guidelines and recommendations for surgery during Covid-19 pandemic: a systematic review. Int J Surg.

[5] Ross SW, Lauer CW, Miles WS, Green JM, Christmas AB, May AK, Matthews BD (2020). Maximizing the calm before the storm: tiered surgical response plan for novel coronavirus (COVID-19). J Am Coll Surg.

[6] ACS guidelines for triage and management of elective cancer surgery cases during the acute and recovery phases of coronavirus disease 2019 (COVID-19) pandemic. American College of Surgeons.

[7] Clinical guide to surgical prioritisation during the coronavirus pandemic. Royal College of Surgeons of England.

[8] Conducting research: when is ethics approval required. National Healthcare Group.

[9] Agha R, Abdall-Razak A, Crossley E, Dowlut N, Iosifidis C, Mathew G, STROCSS Group (2019). STROCSS 2019 guideline: strengthening the reporting of cohort studies in surgery. Int J Surg.

[10] In SM, Park D, Sohn IK, Kim C, Lim HL, Hong S, Jung DY, Jeong S, Han JH, Kim HJ (2015). Experimental study of the potential hazards of surgical smoke from powered instruments. Br J Surg.

[11] de Leeuw RA, Burger NB, Ceccaroni M, Zhang J, Tuynman J, Mabrouk M, Barri Soldevila P, Bonjer HJ, Ankum P, Huirne J (2020). COVID-19 and laparoscopic surgery: scoping review of current literature and local expertise. JMIR Public Health Surveill.

[12] Madhavan S, Shelat VG, Soong S, Woon WWL, Huey T, Chan YH, Junnarkar SP (2018). Predicting morbidity of liver resection. Langenbecks Arch Surg.

[13] Bartlett DL, Howe JR, Chang G, Crago A, Hogg M, Karakousis G, Levine E, Maker A, Mamounas E, McGuire K, Merchant N, Shibata D, Sohn V, Solorzano C, Turaga K, White R, Yang A, Yoon S, Society of Surgical Oncology (2020). Management of cancer surgery cases during the COVID-19 Pandemic: considerations. Ann Surg Oncol.

[14] Chan KS, Ho BCS, Shelat VG (2021). A pilot study of estrogen receptor (ER) expression in pancreatic ductal adenocarcinoma (PDAC). Transl Gastroenterol Hepatol.

[15] Elsamany S, Elbaiomy M, Zeeneldin A, Tashkandi E, Hassanin F, Abdelhafeez N, O Al-Shamsi H, Bukhari N, Elemam O (2021). Suggested modifications to the management of patients With breast cancer during the COVID-19 pandemic: web-based survey study. JMIR Cancer.

[16] Sud A, Jones ME, Broggio J, Loveday C, Torr B, Garrett A, Nicol DL, Jhanji S, Boyce SA, Gronthoud F, Ward P, Handy JM, Yousaf N, Larkin J, Suh Y, Scott S, Pharoah PDP, Swanton C, Abbosh C, Williams M, Lyratzopoulos G, Houlston R, Turnbull C (2020). Collateral damage: the impact on outcomes from cancer surgery of the COVID-19 pandemic. Ann Oncol.

[17] Cancer patient management during the COVID-19 pandemic. The European Society for Medical Oncology.

[18] Mak MH, Chew WL, Junnarkar SP, Woon WW, Low J, Huey TC, Shelat VG (2019). Patient reported outcomes in elective laparoscopic cholecystectomy. Ann Hepatobiliary Pancreat Surg.

[19] Yu H, Chan EE, Lingam P, Lee J, Woon WWL, Low JK, Shelat VG (2018). Index admission laparoscopic cholecystectomy for acute cholecystitis restores Gastrointestinal Quality of Life Index (GIQLI) score. Ann Hepatobiliary Pancreat Surg.

[20] The COVID19 Subcommittee of the O.R. Executive Committee at Memorial Sloan Kettering (2020). Cancer Surgery and COVID19. Ann Surg Oncol.

[21] Saab R, Obeid A, Gachi F, Boudiaf H, Sargsyan L, Al-Saad K, Javakhadze T, Mehrvar A, Abbas SS, Abed Al-Agele YS, Al-Haddad S, Al Ani MH, Al-Sweedan S, Al Kofide A, Jastaniah W, Khalifa N, Bechara E, Baassiri M, Noun P, El-Houdzi J, Khattab M, Sagar Sharma K, Wali Y, Mushtaq N, Batool A, Faizan M, Raza MR, Najajreh M, Mohammed Abdallah MA, Sousan G, Ghanem KM, Kocak U, Kutluk T, Demir HA, Hodeish H, Muwakkit S, Belgaumi A, Al-Rawas A, Jeha S (2020). Impact of the coronavirus disease 2019 (COVID-19) pandemic on pediatric oncology care in the Middle East, North Africa, and West Asia region: a report from the Pediatric Oncology East and Mediterranean (POEM) group. Cancer.

[22] Khalik S (2021). At least one more year of living with COVID-19. The Straits Times.

